# The Development and Validation of an Intercultural Nursing Educator Profile Using the Delphi Method

**DOI:** 10.1177/10436596231207433

**Published:** 2023-11-01

**Authors:** Cinzia Gradellini, Marilize Pretorius, Sofie Vermeiren, Susan Schärli-Lim, Mette Bønløkke, Elena de Lorenzo

**Affiliations:** 1 SUPSI, University of Applied Sciences and Arts of Southern Switzerland (CH)/University of Modena and Reggio Emilia (IT); 2 University of Antwerp, Belgium; 3 University of the Free State, Bloemfontein, South Africa; 4 Artesis Plantijn Hogeschool Antwerpen, Belgium; 5 Zurich University of Applied Sciences, Winterthur, Switzerland; 6 VIA University College, Silkeborg, Denmark; 7 Bioaraba Health Research Institute, Vitoria-Gasteiz, Spain

**Keywords:** cultural competency, nursing education, Delphi technique, professional competence, transcultural nursing, teacher training

## Abstract

**Introduction:**

Educators require focused training to foster the development of intercultural competence in nurses. Training programs for educators need to be based on a comprehensive profile with a focus on intercultural learning. This study aims to define and validate a profile of the Intercultural Nursing Educator (INE).

**Method:**

The Delphi method was used with an iterative, multi-stage process to transform opinions into group consensus. A total of 46 European, African, and American experts from the nursing and intercultural field participated. Inclusion criteria required English at a level of B2, expertise in the field of intercultural competence, experience in teaching intercultural competence in the nursing context, and publications focused on intercultural topics.

**Results:**

The INE profile was developed and all 126 competencies were validated.

**Discussion and conclusion:**

The profile is freely available on the project website and provides the basis for curricula, training programs and assessment of the required competences.

## Introduction

The systematic neglect of culture in health care is the single biggest barrier to the advancement of the highest standard of health care worldwide (Aubel & Chibanda, 2022). Failure to adapt care to a culturally diverse society contributes to an increase in medical errors, length of hospitalization, and avoidable hospitalizations, as well as the over- and under-utilization of procedures, thus widening health care disparities (Smallwood, 2018). The non-centrality of culture in health programs has been shown to be the greatest barrier to care and health progress (Aubel & Chibanda, 2022), and it leads to increased costs in the health care system (Saunders et al., 2017). Neglecting culture means failing to consider patients holistically, thereby increasing inequalities in the way the person is taken care of, is understood, and is informed about access to care.

It is no surprise, then, that the International Council of Nurses (ICN) highlights the need for nurses to be interculturally competent (International Council of Nurses [ICN], 2013; Walkowska et al., 2023). Intercultural competence can be defined as “the communication and behaviour that are both effective and appropriate when interacting across differences” (Deardorff, 2009). Nursing care needs to be based on respect for the cultural characteristics and lifestyles of patients, which means following a culturally competent approach (Červený et al., 2022; Walkowska et al., 2023).

However, research on the level of intercultural competence of nurses indicates that further development of this competence is urgently needed. Training of health professionals has an impact on patient satisfaction, access to health care, and compliance with treatment (Horvat et al., 2014). Yet, nurses range from having an ethnocentric worldview to being moderately interculturally competent (Cai et al., 2021; Červený et al., 2022; Ken Jie et al., 2022; Tosun et al., 2021; Zazzi, 2020). In terms of training in intercultural competence and sensitivity in higher education, a recent review (Gradellini et al., 2021) reveals that there is no uniformity in approaches to such training; thus, the need for a shared international curriculum emerges as well as the need to evaluate the effectiveness of didactic and pedagogic strategies and to invest in expert teacher training.

The necessity of educational programs for students and educators that tend to intercultural competence is evident. The Intercultural Nursing Educator (INE) plays a pivotal role in the complex process of facilitating intercultural competence development. To be effective, the INE should be ethno-relative, meaning that they have a mindset that enables recognition of cultural differences and commonalities, and adaption to others’ cultural backgrounds (Bennett, 2017). In addition, they must be at least one stage ahead of the developmental stage of the students as well as be prepared pedagogically. However, the lack of formal academic preparation of lecturers teaching intercultural competence has been criticized in the past (Long, 2012). Currently, few studies focus on the intercultural competence level of educators (Kirby et al., 2021), although one study indicates moderate levels of intercultural competence (Baghdadi & Ismaile, 2018). The preparedness of nurse educators to facilitate intercultural learning thus remains unclear. It is crucial to create programs to develop INEs’ capacity to train intercultural competence in future nurses.

The international project, Training Intercultural Nursing Educators and Students (TraINErS), financed by Erasmus+ and Movetia aimed to develop such an intervention, namely a program to train INEs. However, first, a profile of INE competences was required. Based on this profile, coherent interventions, such as the one developed in TraINErS, can be created. The first phase of the TraINErS project was thus the development of the INE profile, which is reported on in this article. The aim of this study is to define and validate a profile of the Intercultural Nursing Educator (INE).

## Method

**Figure 1.**
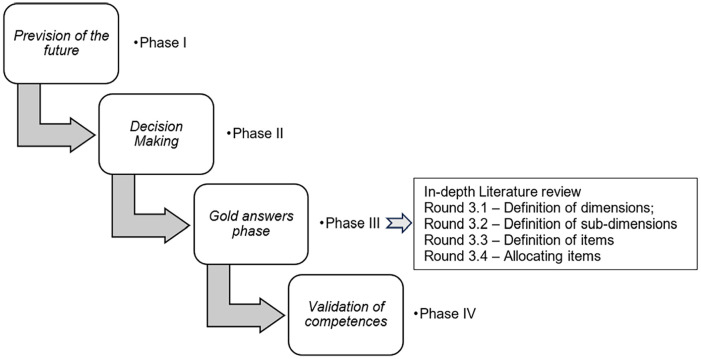
Process of the Delphi Method *Source.* Adapted from McPherson et al. (2018).

This mixed-methods study used an adapted Delphi method (McPherson et al., 2018), which is a variation of the original method (Dalkey & Helmer, 1963). The Delphi method used in this study consists of an iterative, multi-stage process with four phases to transform opinions into group consensus. Hsu and Sandford’s (2007) recommendation for utilizing both quantitative and qualitative methods was followed for data analysis (Figure 1).

In Delphi studies, sample size varies widely based on the purpose and content of the study (Diamond et al., 2014; Trevelyan & Robinson, 2015), but the literature suggests between eight and fifteen participants is optimal (Trevelyan & Robinson, 2015). Given the complexities of using the qualitative approach (i.e., time-consuming process with the need to reduce the risk of participant attrition and keep data processing manageable), a smaller sample is often recommended (Hsu & Sandford, 2007; Trevelyan & Robinson, 2015). In terms of the quantitative approach, the expertise of the participants, based on the study’s parameters and purpose, takes precedence over population representativeness (Hsu & Sandford, 2007; Trevelyan & Robinson, 2015).

### Phase I—Prevision of the Future

The purpose of the first phase was to answer the question: “What are the consequences of the lack of an INE profile”? Seventeen project-partner participants were involved in this phase, as well as for Phase II and III. The project partners were carefully selected to participate in the TraINErS-project based on their expertise; the sample thus constituted a purposive sample and fit recommendations for sample size (Trevelyan & Robinson, 2015). The participants were multi-professional experts in teaching intercultural competence to nursing students in different European countries (see Results). English served as the lingua franca as participants were either sufficiently proficient in English (minimum B2 according to the Common European Framework of Reference [CEFR]) or had another participant available to act as interpreter if necessary. They all had training and experience in teaching intercultural competence in the nursing context and agreed on the theories and frameworks that underpin and inform intercultural competence training. The process consisted of an audio-recorded open discussion. Two external observers followed the discussion and prepared a written report. Two separate researchers then conducted a qualitative semantic analysis to identify the main categories/semantic themes.

### Phase II—Decision-Making

This phase focused on building consensus about the need for an INE profile. The discussion started from the most common competences identified in the literature, as a profile did not seem to exist. After the discussion, each participant responded to the following closed-ended (yes/no) question to confirm consensus: “Do you agree with the need for a definition of a specific profile?” Frequency analysis was used to analyze the data in Phase II.

### Phase III—Gold Answers Phase

This phase aimed to develop a proposal for an INE competence profile and consisted of an in-depth literature review followed by four rounds of seeking consensus, as described below. The criterion for consensus was a minimum of 80% agreement in the answers to the questions on the structure and content of the profile, obtained from frequency analysis.

#### In-Depth Literature Review

No profile stipulating the competences of an INE was found in the literature, requiring an extension of the literature review to identify the required competences. The question we sought to answer through this review was: “What are the competences that should be included in the profile of an INE?” The search was conducted manually in the following databases: Cochrane CENTRAL, MEDLINE, PsycINFO, CINAHL, Proquest Central, and Eric. The search terms were grouped into three constructs: (1) intercultural competency; (2) educators; (3) competences and/or role. The keywords recommended in the thesaurus of each database were combined with the Boolean operators OR and AND. Inclusion criteria were publications such as books, theoretical papers, research articles, or gray literature published in any of the languages of the project-partner countries. Of the 391 texts found, 49 were deemed relevant.

#### Round 3.1—Definition of the Profile’s Dimensions

In this round, decisions were made about the profile construct, using two models. The first proposed construct was a four-dimension model inspired by Horsford et al. (2011), focusing on the Personal, Professional, and Pedagogical Intercultural Competence and Pedagogical Training for Developing Intercultural Competences. The second eligible construct consisted of eight dimensions based on the Nurse Educator Core Competences (World Health Organization [WHO], 2016), focusing on Theories of Adult Learning, Curriculum, Nurse Practice, Evidence-Based Practice, Communication and Partnership, Ethical Principles, Evaluation, Management, and Advocacy. Following an in-depth discussion of the advantages and disadvantages of the models, participants were asked to respond to the question, “Do you prefer the four- or eight-dimension model?”

#### Round 3.2—Definition of the Profile’s Sub-Dimensions

The aim of the second round was to decide on how to organize the four dimensions (Round 3.1) into sub-dimensions. Based on the PISA global competence framework (Program for International Student Assessment [PISA], 2018), three sub-dimensions were proposed: (a) Knowledge; (b) Attitudes and Values; and (c) Skills. The dichotomous question (yes/no) submitted to experts was, “Do you agree on the proposed sub-dimensions?”

#### Round 3.3—Definition of the Profile’s Items

The third round focused on deciding whether the INE competences extracted from the literature review and expert opinion were relevant and should be included. Participants met face-to-face to review and comment on the proposed competences. They subsequently developed a draft of the profile (150 items). Finally, the group responded anonymously (online) to the question, “Is this competence valid?” for each competence statement, to determine whether they are a valid component of the profile. The results were evaluated based on frequencies.

#### Round 3.4—Allocating the Profile’s Items

The aim of this round was to assign the approved competences (Round 3.3) to the dimensions and sub-dimensions decided on in rounds 3.1 and 3.2. The participants were asked to place each competence into the dimensions and sub-dimension anonymously and without comparing their allocations with others. Only one round of questioning was planned and the statistical “mode” was used to assign the competences. The round ended with a syntactic revision of the final profile draft.

### Phase IV—Validation of the Competences

The purpose of Phase IV was to validate the INE profile developed in Phase III. Participants were asked to validate the inclusion and allocation of each competence. In other words, they were asked whether each competence is a valid component of the relevant sub-dimension.

For the external validation of the profile, the participants of the first three preceding phases were excluded. Members of the European Nursing Module network (ENM Network, 2022), a European association that promotes cultural awareness in nursing, were asked to assist in recruiting multinational expert educators. Few nursing educators had formal education in intercultural competence, so to ensure having participants with this expertise, their primary job/role could be in another field, but they were required to have at least a secondary job/role in nursing education to be eligible for recruitment. The experts had to have at least an intermediate level of English (B2) and meet at least two of the following inclusion criteria:

- A minimum of 5 years or 150 hr of experience in teaching inter/transcultural topics (transcultural nursing, anthropology, intercultural communication, or similar topics);
- Five publications or more about intercultural topics;
- Completion of a specific inter/transcultural course with a minimum of 60 ECTS.

Candidates (*n* = 59) received a private e-mail with the informed consent form, demographic questions, and validation survey. The participants had 3 weeks to respond to the survey, which was available via a private link to ensure anonymity and that they were unable to compare responses. The question all 150 competence statements was, “Is this competence valid?.” A six-point Likert-type scale (1 = *totally disagree*; 6 = *totally agree*) was used as it permits the transformation of qualitative data into numbers, allowing for quantitative data analysis (i.e., mean, distribution, and frequencies). With the six-point Likert-type scale (i.e., without a midpoint) a mean ≥3.5 out of 6 was considered significant (Hsu & Sandford, 2007). Also, each section had an open-ended question for suggestions or comments. A second round in Phase IV was planned to re-evaluate items with a mean equal to 3.5 or less, and without 80% agreement.

### Ethical Consideration

All participants—project partners and external experts—signed an informed consent form, which guaranteed confidentiality and anonymization in the event of dissemination of the results. The protocol was approved (SHW_21_05) by the Ethical Committee of the University of Antwerp, which provides ethical clearance to projects conducted at Artesis Plantijn, the coordinator of this project.

## Results

### Description of Participants

The 17 experts of Phases I—III had many years of experience in teaching cultural competence, and came from Belgium, Denmark, Italy, North Macedonia, Spain, and Switzerland. In Phase IV, 29 out of the 59 educators responded, but only 25 (42%) completed the whole survey. This aligns with recommendations for sample size for Delphi studies (Brown, 1968; Dalkey & Helmer, 1963; Hsu & Sandford, 2007; Trevelyan & Robinson, 2015). The sample was 85% female, with 70% older than 45 years, and the rest between the age of 30 and 45. Most participants came from Switzerland (17%), followed by Italy and Belgium (10% each), and then by North Macedonia, the Netherlands and the United Kingdom (7% each). The rest of the participants were from Denmark and France (3% each). About 30% were from “other countries,” namely Germany, Togo, Austria, and the USA. The demographic characteristics of all participants are shown in Table 1.

**Table 1.** Characteristics of the Participants.

Characteristics	Phase I–III		Phase IV	
	*n*	%	*n*	%
Gender				
Male	1	6	4	14
Female	16	94	22	76
Prefer not to answer	0	0	3	10
Age				
30–45	3	18	8	28
46+	14	82	18	62
Prefer not to answer	0	0	3	10
Country				
Italy	2	12	3	10
Belgium	5	35	3	10
Switzerland	4	24	5	17
North Macedonia	1	6	2	7
Denmark	2	24	1	3
Netherlands	0	0	2	7
United Kingdom	0	0	2	7
Serbia	1	6	0	0
Spain	2	12	0	0
France	0	0	1	3
Other^a^	0	0	8	28
Prefer not to answer	0	0	2	7
Discipline of training				
Nursing profession	13	76	11	38
Medical profession	1	6	1	3
Other^b^	3	18	17	59
Prefer not to answer	0	0	0	0
Highest level of education				
Bachelor degree	0	0	4	14
Master degree	12	71	15	52
PhD	5	29	8	28
Prefer not to answer	0	0	1	3
Other	0	0	1	3
Current profession				
In education^c^	14	82	20	69
In clinical practice	0	0	3	10
Other	3	18	5	17
Prefer not to answer	0	0	1	3

a Germany, Togo, Austria, USA. ^b^ Intercultural communication/business administration, Philosophy/Sociology, Management, English language and literature, Education, Public Health, Anthropology. ^c^ University or other higher education.

### Phase I—Prevision of the Future

In the first phase (October 2019), the qualitative semantic analysis concluded with the identification of four related themes:

1. *Effectiveness of intercultural competence training*. The participants agreed that the lack of an INE profile hinders the development of educational programs that are effective or that consider all the necessary competences. This lack also limits the development of tools to assess the educators and the quality of training programs.
2. *Quality of care*. They agreed that the inadequate level of intercultural competence of nurses prevents a person-centered approach to care. Nurses who are ethnocentric do not fully comprehend the significance of patients’ perspectives, experiences, behaviors, or goal, and this affects the relationship and the adherence to treatment.
3. *Access to health care*. The lack of culturally competent nurses indirectly reduces access to health care. Inadequate communication leads to distrust and poor patient assessment. Consequently, patients are unlikely to receive appropriate care and may discontinue treatment.
4. *Health inequities*. The participants argued that the preceding consequences could indirectly result in health inequities, which are a form of discrimination and should be reduced.

### Phase II—Decision-Making

In phase II (October 2019), all partners agreed (100%) on the need for a dedicated INE competence profile. In other words, the need for an INE profile was recognized unanimously.

### Phase III—Gold Answers Phase

#### In-Depth Literature Review

The participants extracted 150 competences related to the profile of an INE from the literature (October 2019—March 2020). The competence statements reflected expertise in the nursing, educational, and intercultural fields, serving as the foundation for the four subsequent rounds.

#### Round 3.1—Definition of the Profile’s Dimensions

The first round resulted in the selection of a 4-dimensional model: Personal, Professional, Pedagogical Intercultural Competence, and Pedagogical Training for Developing Intercultural Competence, with a consensus of 80%. The Personal dimension includes the competences for operating appropriately and effectively in intercultural situations in general. The Professional dimension contains the attributes an interculturally competent nurse needs to provide effective and appropriate care adapted to the cultural background of patients or communities. The Pedagogical Intercultural Competence dimension is comprised of the competences the educator need to foster inclusion in a diverse classroom. The dimension for Pedagogical Training for Developing Intercultural Competence contains competences for facilitating the development of intercultural competence in nursing students and staff.

#### Round 3.2—Definition of the Profile’s Sub-Dimensions

In the second round, the competence statements were classified into the 3 sub-dimensions with a 90% agreement (15 out of 17 participants). The sub-dimensions, from the PISA (2018) global competence framework, are (a) Knowledge, (b) Attitudes and Values, and (c) Skills.

#### Round 3.3—Definition of the Profile’s Items

Round three reduced the initial 150 INE competence statements to 126 statements. This was achieved by either deleting statements or integrated them into other competences.

#### Round 3.4—Allocating the Profile’s Items

In the last round, the allocation of the competences to the four dimensions and three sub-dimensions was completed using the most frequent proposals (mode). The resulting first draft of the profile consisted of 35 competences in knowledge, 34 in attitude and values, and 57 in skills (Table 2).

**Table 2:** Organization of the Competences/Items in Dimensions and Sub-Dimensions.

Dimension	Personal			Professional			Pedagogical intercultural competent			Pedagogical training intercultural competence			Total
Sub-dimension	K	A/V	S	K	A/V	S	K	A/V	S	K	A/V	S	
Number of items per sub-dimension	6	10	16	12	12	11	9	7	12	9	5	17	126
Total number of items per dimension	32			35			28			31			126

*Note*. K = knowledge; A/V = attitudes & values; S = skills.

### Phase IV—Validation of the Competences

In the final phase (April 2020), all competencies were validated based on the agreement of all participants. Regarding the Personal Intercultural Competence, all items in the three sub-dimensions are valid. Even the items with the lowest means in each sub-dimension (i.e., item 3 in *Knowledge* [*M* = 5.16; *SD* = 0.80]; item 15 in *Attitude & Values* [*M* = 5.36; *SD* = 0.75]; item 20 in *Skills* [*M* = 5.04; *SD* = 1.11]) had means higher than the acceptable mean of 3.5 (see items in bold in Table 3).

**Table 3:** Agreement on Personal Intercultural Competence.

**Question**—The educator is able to	*n*	Mean
Knowledge		
1 . . . describe what culture is and recognize elements of culture which affect intercultural interaction	25	5.60
2 . . . explain the influence perception plays on the attribution of meaning	24	5.56
3 . . . explain sociolinguistic awareness, specifically intercultural communication styles and local language	25	5.16
4 . . . describe deep cultural knowledge related to self and cultural others	25	5.36
5 . . . recite cultural frameworks for exploring cultural value differences	25	5.40
6 . . . formulate a grasp of global issues	25	5.20
Attitudes & values		
7 . . . shows respect and values the cultural other	25	5.96
8 . . . shows openness to new experiences and to people who are different	25	5.92
9 . . . demonstrates curiosity—interest in seeking out cultural interactions and the cultural other	25	5.80
10 . . . tolerates ambiguity	24	5.54
11 . . . recognizes cultural self-awareness	24	5.68
12 . . . shows patience	25	5.68
13 . . . responds with sense of humility	25	5.40
14 . . . shows global mindedness	25	5.44
15 . . . gives everyone the benefit of the doubt and thereby assume the intentions of others are positive	25	5.36
16 . . . values human dignity and diversity	25	5.84
Skills		
17 . . . show listening, observing and evaluating skills using patience and perseverance to unlock meaning	25	5.56
18 . . . show behavior that demonstrates challenging the own cultural assumptions	25	5.52
19 . . . engage with cultural others; seeks cooperation and involvement	25	5.68
20 . . . implement intercultural conflict management and resolution	25	5.04
21 . . . develop multiple frames of reference for interpreting and analyzing intercultural interactions	25	5.24
22 . . . communicate effectively and appropriately with cultural others	25	5.68
23 . . . understand the perspectives of others’ ideas before responding; watch and wait; clarify and paraphrase to achieve shared meaning	24	5.54
24 . . . suspend judgment long enough to examine multiple perspectives and interpret behavior (suspend the automatic pilot)	24	5.63
25 . . . show cognitive and behavioral flexibility	24	5.71
26 . . . utilize cultural frameworks to become more self-aware and observe cultural patterns	25	5.56
27 . . . reflect on the meaning of their intercultural encounters	23	5.65
28 . . . select from a broad repertoire of behavior which is appropriate to the cultural context and the desired outcome	25	5.32
29 . . . differentiate between cultural stereotyping and cultural generalizations and demonstrate the ability to formulate generalizations as a working hypothesis	25	5.48
30 . . . practise cultural bridging	25	5.52
31 . . . maintain perception of both commonalities and differences across cultures	25	5.52
32 . . . accept and respect cultural values of others	25	5.64

*Note.* Likert-type scale (1/*totally disagree*; 6/*totally agree*).

All the items reached a 6 (*totally agree*) on the Likert-type scale as mode. In this dimension, all items had a mean higher than the minimum of 3.5. For 31 items out of 32, the mode corresponds to 6 (*totally agree*) on the Likert-type scale, while the remaining one item corresponded to a 5 (*agree*).

Some comments (6) were made in the space provided for this purpose. Four of them specify that all the proposed items are relevant, but should be considered indicative of an “ideal educator” in terms of being a top performer. For example, one participant commented the following: “Practically all of these are very relevant, the issue here is probably that this would refer to an ideal educator, who in reality may still have to further develop some of these skills.” This is an important caveat for the application of this profile—it reflects a high level of competence which requires on-going training and development.

Regarding Professional Intercultural Competence, in all three sub-dimensions, the items all scored well above the 3.5 cut-offs (see items in bold in Table 4). In *Knowledge*, the lowest mean was for the competence statement in Item 37 (*M* = 5.26; *SD* = 0.85), in *Attitude & Values* the lowest mean was for Item 47 (*M* = 5.05; *SD* = 0.93), and in *Skills* three items shared the lowest mean (*M* = 5.38): Item 58 (*SD* = 0.9); Item 59 (*SD* = 0.7); and Item 60 (*SD* = 0.81). All the items had a mean higher than the minimum of 3.5. For 34 items out of 36, the mode corresponds to 6 (*totally agree*) on the Likert-type scale, while two items correspond to 5 (*agree*).

**Table 4.** Agreement on Professional Intercultural Competence.

Knowledge		
**Question—**The educator is able to	*n*	Mean
33 . . . describe the meaning of culture and the theories on cultural learning and intercultural competence	23	5.39
34 . . . explain the diversity of health beliefs, of patient, family and nurse roles and their mutual expectations	23	5.52
35 . . . understand the stages from ethnocentrism to ethnorelativism	22	5.27
36 . . . describe ethical dilemmas about cultural diversity and the determinants in health inequalities	22	5.65
37 . . . demonstrate knowledge about cultural shock	23	5.26
38 . . . understand migratory and acculturation processes	23	5.30
39 . . . understand culture and care practices of other society and cultural groups and health belief models	23	5.52
40 . . . understand the emotional experience, thoughts and behaviors of persons with a different cultural background	23	5.74
41 . . . describe the principles of intercultural communication	21	5.48
42 . . . explain the tools to perform nursing assessments sensitive to culture	23	5.61
43 . . . describe intercultural conflict prevention and management of racism, prejudice, and stereotypes as unconscious bias	23	5.39
44 . . . describe strategies to adapt educational and care intervention to culture	21	5.29
Attitudes & Values		
45 . . . demonstrates awareness of own culture and individual worldview toward cultural difference and its effect on care	24	5.71
46 . . . demonstrates awareness of stereotypes, prejudices, and cultural biases	24	5.79
47 . . . demonstrates cultural desire	22	5.05
48 . . . demonstrates a genuine and sincere motivation to increase intercultural competence	24	5.50
49 . . . demonstrates cultural empathy and approaches differences sensitively	24	5.83
50 . . . expresses cultural humility	24	5.46
51 . . . shows the desire to correct power imbalances	24	5.46
52 . . . shows interest in knowing the culture of the student or patient	24	5.75
53 . . . believes that no culture is superior to others	24	5.92
54 . . . demonstrates commitment to teach and provide care sensitive to culture	24	5.88
55 . . . demonstrates open-mindedness and flexibility	24	5.93
56 . . . accepts and respects different ways of doing and seeing the world	24	5.88
Skills		
57 . . . show intercultural knowledge in terms of skills	24	5.46
58 . . . use tools to perform nursing assessments adapted to culture	24	5.38
59 . . . help in the acculturation process of people newly immersed in another culture	24	5.38
60 . . . manage intercultural care and understanding individual life-worlds in specific situation and in various contexts and to infer appropriate ways of action from this boundary situations	24	5.38
61 . . . manage intercultural communication skills	23	5.57
62 . . . use strategies to prevent and manage intercultural conflict	24	5.42
63 . . . apply social equity in care	24	5.54
64 . . . analyze and manage ethical dilemmas that emerge from cultural diversity	24	5.63
65 . . . manage prejudice	24	5.58
66 . . . work in a multidisciplinary team	23	5.57
67 . . . provide culturally congruent and sensitive care	24	5.58

*Note.* Likert-type scale (1/totally disagree; 6/Totally agree).

A few comments (6) were also made. Overall, the comments emphasize the value of these competences, for example: “This part is excellent. It had all the necessary elements of the skills of an educator.” The participants thus overwhelmingly agreed that the knowledge, skills and the values/attitudes reflected in this dimension were necessary and relevant. Nevertheless, even though the mean was higher than expected, two suggestions from the comments were adopted. The item, “show intercultural knowledge into skills,” has been changed to “show intercultural knowledge in skills.” Also, the competence statement, “understand relevant laws and human rights related to migrants,” has been added to the *Knowledge* sub-dimension.

In the dimension for Pedagogical Intercultural Competence (Table 5), again all items reached more than the minimum 3.5 mean in all three sub-dimensions. In the sub-dimension *Knowledge*, item 71 had the lowest mean (*M* = 5.17; *SD* = 1.01), in *Attitude & Values* item 79 had the lowest mean (*M* = 5.52; *SD* = 0.58), and in the sub-dimension *Skills*, the lowest mean was 5.25 (*SD* = 0.83) for item 84. For all items the mode corresponds to 6 (*totally agree*) on the Likert-type scale.

**Table 5.** Agreement on Pedagogical Intercultural Competence: Intercultural Competent Educator.

Knowledge		
**Question—**The educator is able to	*n*	Mean
68 . . . support the students in recognizing and explaining the impact of their own culture on their learning process, group dynamics, relationship building, their communication and conflict style	23	5.52
69 . . . analyze the cultural factors that condition learning	23	5.35
70 . . . associate how culture influences the style of learning, thinking, and communicating	23	5.65
71 . . . formulate the impact of the culture of the educator on the content they teach	23	5.17
72 . . . describe the impact of culture on the trainer-learner relationship and on the learner- learner relationship e.g. power, role-modeling, source of expertise	23	5.43
73 . . . identify risk factors, challenges and barriers that learners might surface during teaching activities	23	5.30
74 . . . explain the principles of intercultural communication to enhance the teaching and learning process	23	5.48
75 . . . describe educational strategies to lead a culturally diverse group	23	5.22
76 . . . compare local, regional and international ethical codes of conduct and obligations related to nursing education and practice	23	5.26
Attitudes & Values		
77 . . . acknowledges, accepts and integrates cultural differences among learners or educators and various ways of learning	23	5.67
78 . . . demonstrates a genuine motivation to approach a cultural other	23	5.65
79 . . . demonstrates a positive attitude to providing pedagogical interventions adapted to the culture of the student	23	5.52
80 . . . feels cultural empathy for students` need of safety and trust	23	5.83
81 . . . recognizes power differences, and allows for cultural variability in the classroom, educator-student/learner-learner	23	5.57
82 . . . demonstrates commitment toward the promotion of ethical behaviors of respect, dignity, open-mindedness, and tolerance of the cultural other	23	5.78
83 . . . displays honesty and fairness in all monitoring activities	23	5.57
Skills		
84 . . . recognize and validate cultural differences in writing and communication	24	5.25
85 . . . provide educational interventions adapted to cultural diversity	24	5.42
86 . . . demonstrate empathy in situations with culturally diverse students	24	5.63
87 . . . relativize own values, norms and expectations concerning education	23	5.57
88 . . . show inclusiveness where all feel connected and respected	24	5.71
89 . . . model ethical behavior of respect, dignity, open-mindedness, acceptance of ambiguity and of the cultural other	24	5.67
90 . . . use intercultural communication skills in the teaching and learning process	24	5.67
91 . . . use strategies to manage intercultural conflict in the teaching and learning process	24	5.54
92 . . . model and encourage perspective shifting/multi-perspectives in the teaching and learning process	24	5.42
93 . . . apply social justice in education, analyzing the ethical dilemmas that emerge from cultural diversity	23	5.57
94 . . . be self-reflective as an educator (e.g. about their own interpretations, unconscious bias, pre-justice, and stereotyping . . .)	23	5.83
95 . . . create opportunities for peer learning and interaction among diverse learners	23	5.74

*Note.* Likert-type scale (1/*totally disagree*; 6/*totally agree*).

Similar results were obtained for the dimension Pedagogical Training Intercultural Competences, with all items having means well above 3.5. In Table 6, you can see that in the sub-dimension *Knowledge*, 5.00 (*SD* = 0.82) was the lowest mean (item 99). In *Attitude & Values*, item 105 had the lowest mean (*M* = 5.39; *SD* = 0.82). The lowest mean (*M* = 5.00; *SD* = 0.88) in the sub-dimension *Skills* was for Item 113. In 27 out of 31 items, the mode corresponds to 6 (*totally agree*) on the Likert-type scale, and the remaining items correspond to a 5 (*agree*).

**Table 6.** Agreement on Pedagogical Intercultural Competence: Educator Who Trains Intercultural Competence.

Knowledge		
**Question—**The educator is able to	*n*	Mean
96 . . . develop goals, objectives, and content of the educational program for the development of cultural competence in nursing	23	5.35
97 . . . identify the relevant pedagogical perspectives and theories to train intercultural competence	23	5.43
98 . . . describe conceptual frameworks that present the continuum of development of intercultural sensitivity (e.g.: Developmental Model of Intercultural Sensitivity)	23	5.26
99 . . . explain the purpose, advantages and disadvantages of the different experiential and didactic training strategies, according to the student stage of development of cultural sensitivity	23	5.00
100 . . . describe (risk)factors such as cultural shock, stress, resistance, and denial that affect learning for students developing intercultural competence	23	5.22
101 . . . explain the affective-emotional reactions and the coping strategies of the students that are in the process to develop intercultural competence	23	5.17
102 . . . describe the principles of intercultural communication and theories of intercultural relations	23	5.43
103 . . . describe the principles of coaching for students in a cultural immersion	22	5.18
104 . . . explain the advantages and disadvantages of the different methodologies and tools to assess and evaluate cultural competence programs	23	5.04
Attitudes & Values		
105 . . . shows commitment toward the continuous adaptation of the teaching and learning strategies to individualize the learning process to develop intercultural competence	23	5.39
106 . . . shows a positive attitude toward applying active and innovative methods aimed at reflective and critical thinking to develop intercultural competence	23	5.65
107 . . . demonstrates commitment toward the ethics of culture teaching and learning and the ethics of culture contact	23	5.61
108 . . . demonstrates caring, integrity, mutual trust, respect, enthusiasm, patience, tolerance with ambiguity, and flexibility to facilitate learning	23	5.70
109 . . . demonstrates commitment to life-long learning about intercultural competence and the way to train it	23	5.65
Skills		
110 . . . use conceptual frameworks needed for the development of intercultural competence	23	5.52
111 . . . design integrated training programs with the appropriate mix of experiential and didactic methods, culture-specific and culture-general content, and cognitive-affective-behavioral-learning activities	23	5.30
112 . . . use appropriate methods to assess the readiness and intercultural competence of the students to sequence interventions appropriately and to individualize their training	23	5.13
113 . . . utilize specific strategies for each developmental stage to enhance intercultural development	23	5.00
114 . . . use motivational strategies throughout the learning sequences to enhance effective learning	23	5.26
115 . . . foster reflection on differences and commonalities and analysis to understand the impact of culture, and the dangers of ethnocentrism in nursing	23	5.61
116 . . . help learners to suspend judgment long enough to examine multiple perspectives—to stop the automatic pilot	22	5.55
117 . . . induce the use of multiple frames of reference for interpreting intercultural situations	23	5.48
118 . . . facilitate strategies to enhance the awareness of the students` own culture construction	22	5.50
119 . . . model and guide the exploration and practice of perspective shifting	22	5.45
120 . . . induce the analysis of the potential ethical issues and dilemmas in relation to the care of the different	23	5.43
121 . . . guide, challenge and support intercultural students effectively	23	5.48
122 . . . employ counseling strategies that help students to deal with cultural challenges, to recognize their sources of stress, to help them make sense of the experiences and to manage their emotional reactions	23	5.52
123 . . . use appropriate methods of evaluation of the level of intercultural competence of the students	23	5.30
124 . . . analyze the ethical dilemmas of the impact on students of the teaching-learning process to promote cultural competence	22	5.32
125 . . . create and maintain a safe environment that is conducive to learning intercultural competence	23	5.74
126 . . . foster teamwork and collaboration with educational and clinical institutions at the local, national, and international level	23	5.48

*Note.* Likert-type scale (1/totally disagree; 6/Totally agree).

A second round to re-evaluate items was unnecessary, as there were no items below the 3.5 cut-off point. After the analysis, participants were contacted by e-mail, asking whether they wanted to adjust any of their initial ratings (Diamond et al., 2014). However, no requests for changes were made.

## Discussion

The profile was enriched by the international, multicentric, and multi-professional nature of the sample, with participants having extensive experience teaching in higher education. It also included a voice from the global south. The combination of methods contributes to the methodological rigor of this study.

Some limitations must be noted, though. The sample could be considered small, but is larger than what the literature suggests for the Delphi Method (Brown, 1968; Dalkey & Helmer, 1963; Diamond et al., 2014). Indeed, a benefit of the sample was the representation of experts in both nursing and intercultural competence, allowing for a comprehensive perspective. The participants also met the academic and professional requirements. Few participants had advanced academic qualifications (MA, MSc & PhD) in the intercultural field and the discussions in the project might have been enriched by more participants with formal education in this topic. However, a lack of advanced intercultural training in nursing is a broader reality (Long, 2012). The response rate in Phase IV (42%) is somewhat low but understandable given the length of the survey, the 3-week response deadline, and the many demands on experts’ time.

Nevertheless, the experts tended to strongly agree with the relevance of and necessity to include the competences in the profile, as well as the (sub-)dimension allocation of the 126 items, resulting in only one round of quantitative validation as consensus was reached (Diamond et al., 2014). Also, the competences in this profile reflect an advanced level of competence. In the TraINEr-Sproject, the Rubric Assessment of Intercultural Nursing Education Development (RAINED; https://trainers.ap.be/the-trainers-projects-outcomes/) was developed to measure pre- to post-training development. However, further research is required to determine cut-off points to differentiate between levels of competence.

A strength of the profile is its broad applicability. Though the profile was created for nursing, it could be used in other health care professions. Many of the competences can be applied directly, but some would require a degree of adaptation. The profile is in basic English, but when translating, project partners encountered difficulties. Validation of the profile in each language is thus recommended.

The validated INE profile is an invaluable basis for designing educational interventions. Education must track with social, cultural and global health changes, recognizing the need for cultural competence as an essential element of safe (Walkowska et al., 2023) and high-quality care (Ferreira Aydogdu, 2022). Culturally competent nurses can respond to global change by listening and responding in an individualized way to the diverse demands that are becoming pervasive in nursing (Tosun et al., 2021; Walkowska et al., 2023). Yet nurses are less ethno-relative than many other professionals (Zazzi, 2020), and calls to invest in educational pathways to cultural competence remain unheeded (Ken Jie et al., 2022). Few proposed educational interventions have demonstrated their effectiveness, instead revealing inconsistent standards for content and methodology (Červený et al., 2022; Tosun et al., 2021). This may result in reduced opportunities to train and subsequently practice culturally competent care (Červený et al., 2022; Tosun et al., 2021).

The profile serves as a training blueprint for turning nurse educators into INEs. It can (or should) be used to create professional development programs or in-service training, with an initial focus on personal and professional development. In other words, being ethno-relative at the personal level is foundational to performing adequately as both a nurse and an educator. Also, cultural competence should not be understood as an outcome, but as an ongoing developmental process (Walkowska et al., 2023). Self-awareness becomes fundamental to promoting understanding, acceptance, and respect for others as bearers of difference (Walkowska et al., 2023).

Nursing students need pedagogically competent educators who can use experiential learning and guided reflection to facilitate the process of becoming interculturally aware and develop intercultural competence (Lou & Bosley, 2012; Mikkonen et al., 2016; Vande Berg et al., 2012). Thus, nurse educators should be trained to plan and implement educational interventions based on a competence profile, such as the INE profile, to make informed pedagogic and didactic decisions in selecting content, teaching methods, and assessment. Considering that the literature underlines a lack of uniformity in the programs for developing intercultural competence as part of nursing curricula (Gradellini et al., 2021), this profile has been developed with an international perspective so that it can be used as a guideline to develop coherence and consistency across nursing curricula.

If the literature emphasizes the need for culturally competent nurses (ICN, 2013; Walkowska et al., 2023), it is clear the need to invest in their basic and continuing education. Students must be guided by educators who are themselves interculturally competent (Ferreira Aydogdu, 2022; Walkowska et al., 2023). Teacher training is essential, since, as catalysts for change, they should be experts in the issues addressed (Gradellini et al., 2021). If the educator is the facilitator of the learning process (Vande Berg et al., 2012), then we need to ensure that the educators have the necessary competences to develop the intercultural competence of students as future nurses. Given the complexity and multidimensionality of each individual patient and the moderate levels of specific intercultural competence of nurse educators (Baghdadi & Ismaile, 2018; Long, 2012), it is necessary to start from the competence of educators, a theme that until now has received little attention in the literature (Kirby et al., 2021). The use of the INE profile should result in making intercultural competence training an integral part of undergraduate core curricula, with the aim of producing personally and professionally competent nurses.

## Conclusion

A dedicated, validated profile for INEs is now freely available, addressing a serious gap in the literature. This profile will only provide real value when transformed into professional practice. The TraINErS- project therefore used this profile to develop a theoretically sound (blended) training program consisting of online materials to train educators involved in the development of the intercultural competence of students. The training program will be freely available online by the end of 2023 (https://trainers.ap.be/the-trainers-projects-outcomes/). The use and possible expansion of this program would contribute to developing culturally competent nurse educators, thereby improving intercultural learning in nurse education programs and increasing the level of intercultural competence of nurses. Hopefully, in the long term, it will have an impact on the access and quality of health care, resulting in an increase of social equity.
